# Breed Selection of Poplars Imposes Greater Selection Pressure on the Rhizosphere Bacterial Community

**DOI:** 10.3390/microorganisms12112176

**Published:** 2024-10-29

**Authors:** Jinliang Liu, Long Zhou, Yan Lan, Junfeng Fan

**Affiliations:** 1 Key Laboratory of Silviculture on the Loess Plateau State Forestry Administration, College of Forestry, Northwest A&F University, Xianyang 712100, China; liujinliang2016@nwafu.edu.cn (J.L.); zlong@nwafu.edu.cn (L.Z.);; 2 Forest Ecosystem National Observation and Research Station of Huanglong Mountains, Yanan 715700, China

**Keywords:** breed selection, rhizosphere microbiomes, untargeted metabolomics, poplar

## Abstract

Breed selection alters the coevolution of plant–microbiome associations that have developed over long periods of natural evolution. We investigated the effects of breed selection on the rhizosphere microbiomes and metabolites of hybrid parents (I101 and 84K) and their offspring (Q1–Q5) using metagenomics and untargeted metabolomics. Rhizosphere archaeal, bacterial and fungal community β-diversity significantly differed among hybrid parents and offspring, but only the dominant bacterial phyla and bacterial community α-diversity revealed significant differences. Approximately 5.49%, 14.90% and 7.86% of the archaeal, bacterial and fungal species significantly differed among the poplar hybrid parents and offspring. Rhizosphere microbial functional genes and metabolites were both clustered into the following three groups: I101 and 84K; Q2 and Q4; and Q1, Q3 and Q5. Compared with the hybrid parents, 15 phytochemical compounds were enriched in the hybrid offspring and explained 7.15%, 18.24% and 6.68% of the total variation in the archaeal, bacterial and fungal community compositions, respectively. Rhizosphere metabolites significantly affected the bacterial community, rather than the archaeal and fungal communities. Our observations suggested that poplar breed selection imposed greater selection pressure on the rhizosphere bacterial community, which was mainly driven by metabolites.

## 1. Introduction

Breed selection is a process of selecting more productive and suitable plant genotypes that can adapt to different environments [1,2]. Breeding accelerates plant evolution, which in turn changes the coevolution of plant–microbiome associations [1]. The rhizosphere microbiome associated with plant roots is referred to as the second genome of the plant and it can contribute to nutrient acquisition, pathogen protection, stress tolerance and phenotypic plasticity in plants [3,4,5,6]. The substantial impact of beneficial microorganisms on plant health and growth has prompted breeders to pay attention to plant–microbiome interactions during the breeding process [7,8]. Breeding strategies based on plant–microbiome interactions have been considered in crop breeding [2]. However, the influence of breed selection on the rhizosphere microbiome of woody plants is poorly understood.

Rhizosphere microbiomes can form complex co-associations with plants and are recognized as an extension of the plant’s root phenotype [3,9]. Root-associated microbiomes directly affect host plants, including by the transformation and translocation of nutrients, mitigation of environmental stress and protection from plant pathogens [9]. Colonization by mycorrhizae can lead to generalized metabolic changes and improved N and P absorption in plants [10] and a single bacterial genus (*Variovorax*) can manipulate plant hormone levels to maintain root growth [4]. Moreover, root-associated microbiomes can also indirectly contribute to host plants by enhancing the resistance responses of plants via interactions between the plants and microbiomes that coregulate plant traits [9].

Rhizosphere microbiomes are mainly controlled by root exudates, given that 11% of the net fixed C is exuded to the rhizosphere by roots [10,11,12]. Root exudates are low-molecular-weight compounds and consist of an assortment of primary metabolites and secondary metabolites such as organic acids, amino acids, terpenoids and phenolics [10,13]. These complex chemical substances can change the rhizosphere microenvironment and act as nutrient sources and signals to affect the recruitment and assembly of rhizosphere microflora [5,11,13,14,15]. Some phytohormones, such as ethylene, abscisic acid, salicylic acid and jasmonic acid, play important roles in mycorrhizal colonization and some microorganisms need the presence of root exudates to survive [10]. In addition to root exudates, habitat and management practices also play important roles in the assembly and functions of rhizosphere microbiomes [16].

Root exudates are not the only source of rhizosphere metabolites, which consist of complex compounds secreted by roots and the organisms that interact with the roots [17]. Untargeted metabolomics is one of the key technologies used to identify small molecules in the rhizosphere and to understand the functional role of microbial metabolites [18]. Metabolic-pathway-based approaches can link metabolites to biochemical pathways and provide insights into the function of microbial communities [18,19]. Integrated multiomic analyses, including soil metabolomics and microbial metagenomics, provide a better understanding of the interactions between plants and microbiomes [20].

The differences in rhizosphere microbial compositions increase with an increase in phylogenetic distance and intraspecific genotypic variations can significantly affect the phylogenetic structure of root-associated microbiota [16,21]. Mounting evidence has demonstrated that root-associated microbiomes dramatically change with long-term breeding [8]. There is a growing consensus that the selection, development and application of beneficial associated microbiomes facilitates the development of next-generation plant-breeding strategies [2,5,7]. Poplar is a major industrial tree species in China and has great potential for industrial use and renewable bioenergy production [22]. In the present study, we analyzed the rhizosphere microbiome and metabolites using metagenomic and untargeted metabolomic methods. The aims of this work were (i) to explore the effects of breed selection of poplars on the rhizosphere microbial community composition, structure and function and on rhizosphere metabolites, and (ii) to uncover the effects of metabolites on rhizosphere microbiomes.

## 2. Materials and Methods

### 2.1. Poplar Breed Selection and Study Site

A poplar hybrid (I101 (*Populus alba* L.) × 84K (*Populus alba* L. × *Populus glandulosa* Uyeki)) was used to breed new hybrid varieties that exhibited fast growth, strong adaptability, easy reproduction and climate adaptation in the northwestern China. After artificial hybridization and breeding, QinBaiYang hybrid offspring (Q1~Q5) were selected. To date, Q1–Q3 and Q5 have passed the examination and approval of the forest tree varieties of China and Q4 has passed the examination and approval of the forest tree varieties of Shaanxi Province.

This study was carried out at the Weihe Research Station (108°17′22″ E, 34°11′45″ N) of Northwest Agriculture and Forestry University in Shaanxi Province, China. The site has sandy loam soil and a warm temperate continental monsoon climate with a mean annual rainfall of 650 mm and a temperature of 13.3 °C. An experimental forest of poplar hybrid parents (I101 and 84K) and their offspring (Q1~Q5) was established using a randomized complete-block design with 5 replicate plots in March 2018. The plant spacing and row spacing were 4 m × 4 m and a total of 25 trees were planted for each poplar variety in 5 replicate plots. The same soil type, climate and forest management practices ensured that the variation in the rhizosphere soil microbial community was mainly driven by the poplar hybrid parents and their offspring.

### 2.2. Sample Collection and Soil Nutrient Analysis

Rhizosphere soil samples were collected from the topsoil layer (0–20 cm) 1–1.5 m away from the tree using a stainless-steel corer 10 cm in diameter in July 2022. In each replicate plot, four trees with similar diameters at breast height (DBHs) and heights were selected for each poplar variety and four rhizosphere soil samples from four trees were mixed together into one soil sample. A total of 35 rhizosphere soil samples were collected from 5 replicate plots. In this study, rhizosphere soil was isolated from roots with diameters less than 2 mm. The soil that was manually shaken from the selected roots was used to analyze soil nutrients and the remaining roots were cut into 4 cm segments using sterile scissors and stored in a 50 mL tube in liquid nitrogen. These roots were transported to the laboratory and placed in a sterile tube with 40 mL of a sterile phosphate-buffered saline (PBS) solution. Soils attached to the root surface were cleaned using a sonication protocol and the sterile tube was sonicated for 30 s in the range of 50–60 Hz. After removal of the roots, the liquid PBS fraction was centrifuged at 6000× *g* for 20 min at 4 °C [23]. The soil was then collected and stored at −80 °C for metagenomic and untargeted metabolomic analyses. Soil nutrients such as soil organic carbon (SOC), total nitrogen (TN), total phosphorus (TP), nitrate nitrogen (NO_3_^−^), ammonium nitrogen (NH_4_^+^) and available phosphorus (AP) were quantified as previously reported [24].

### 2.3. Soil DNA Extraction and Metagenomic Sequencing

Soil total DNA was extracted using an E.Z.N.A.^®^ Soil DNA Kit (Omega Bio-Tek, Norcross, GA, USA) according to the manufacturer’s instructions. The concentration, purity and quality of the extracted DNA were checked using a TBS-380, a NanoDrop2000 and 1% agarose gel electrophoresis. Covaris M220 (Gene Company Limited, Shanghai, China) was used to fragment the extracted DNA to an average size of approximately 400 bp and NEXTflex™ Rapid DNA-Seq (Bioo Scientific, Austin, TX, USA) was used to construct the paired-end library. Paired-end sequencing was performed using the Illumina NovaSeq/HiSeq Xten platform (Illumina, Inc., San Diego, CA, USA) by Majorbio Bio-Pharm Technology (Shanghai, China). The raw reads were cleaned by removing adaptor sequences and low-quality reads and the optimized reads were assembled into contigs using MEGAHIT (v1.1.2) [25]. Contigs with lengths less than 300 bp were removed and the open reading frames of the remaining contigs were identified and clustered using MetaGene 2 and CD-HIT (v 4.6.1) [26,27]. Based on the nonredundant gene catalog with 95% identity, the gene abundance in each soil sample was determined using SOAPaligner (soap2.21release) [28]. Annotation information of nonredundant genes and the abundance of species was obtained using Diamond (v 0.8.35), HMMER (v 3.1b2), the NCBI NR database and Kyoto Encyclopedia of Genes and Genomes database (KEGG) [29].

### 2.4. Untargeted Metabolomic Profiling

The soils were extracted using 4:1 (*v*/*v*) methanol–water, ground while frozen for 6 min (−10 °C; 50 Hz), subjected to low-temperature ultrasonic extraction for 30 min (5 °C; 40 kHz), left at −20 °C for 30 min and centrifuged for 15 min (4 °C; 13000 × g). The supernatant was transferred to sample vials for a liquid chromatography/mass spectrometry (LC-MS) analysis. The LC-MS analysis was conducted using a Thermo UHPLC-Q Exactive HF-X system equipped with an ACQUITY HSS T3 column (100 mm × 2.1 mm i.d.; 1.8 μm) (Waters, Milford, MA, USA) and an electrospray ionization (ESI) source at Majorbio Bio-Pharm Technology Co., Ltd. (Shanghai, China). The mobile phases consisted of 0.1% formic acid in water–acetonitrile (95:5, *v*/*v*) and 0.1% formic acid in acetonitrile–isopropanol–water (47.5:47.5:5, *v*/*v*/*v*), with a flow rate of 0.40 mL/min and a column temperature of 40 °C. The mass spectrometric conditions were set as follows: source temperature of 425 °C; sheath gas flow rate of 50 arb; aux gas flow rate of 13 arb; and floating ion-spray voltage at −3500 V in negative mode and 3500 V in positive mode. The full MS and MS/MS resolutions were 60,000 and 7500, respectively. Data acquisition was performed using the data-dependent acquisition (DDA) mode and detection was carried out over a mass range of 70–1050 *m*/*z*. The pretreatment of the raw LC-MS raw data was performed using Progenesis QI (v2.0) software (Waters Corporation, Milford, CT, USA). Internal standard peaks and any known false-positive peaks were removed from the data matrix and the remaining peaks were then subjected to a de-redundancy analysis and pooled. The metabolites were identified using HMDB (http://www.hmdb.ca/, accessed on 20 December 2022) and Metlin (https://metlin.scripps.edu/, accessed on 20 December 2022).

### 2.5. Statistical Analysis

The variations in the soil microbial community composition, diversity and soil nutrients among the poplar varieties were analyzed using a one-way analysis of variance (ANOVA) and a least significant difference (LSD) multiple comparison test. The β-diversity of the rhizosphere soil bacterial, fungal and archaeal communities was identified by nonmetric multidimensional scaling (NMDS) using the ‘vegan’ package [30]. A hierarchical clustering analysis with a heatmap was used to explore the similarity of microbial functional genes and metabolites among hybrid parents and offspring. A linear discriminant analysis effect size (LEfSe) analysis of microbial community compositions, a heatmap analysis and an enrichment analysis of KEGG level 3 pathways were performed using the online platform of the Majorbio Cloud platform (www.majorbio.com, 24 October 2024). Bacterial species with relative abundances greater than 0.01% and archaeal and fungal species with relative abundances greater than 0.05% were selected for a co-occurrence network analysis using the ‘igraph’ package based on Pearson’s correlation coefficients (*p* < 0.05; r ≥ 0.9), after which visualization was performed using Gephi-0.9.2 [31]. The co-occurrence network analysis of microbial functional genes associated with N and P cycles was also performed using the ‘igraph’ package based on Pearson’s correlation coefficients (*p* < 0.05; r ≥ 0.8), after which visualization was performed using Gephi-0.9.2 [31]. A volcano plot was used to analyze the variations in metabolites among different clustered poplar groups. The variable importance in projection (VIP) score was used to analyze the variations in phytochemical compounds among cluster groups. The effects of soil nutrients on dominant microbial phyla, diversities and compositions (species level; relative abundance > 0.05%) were analyzed using a multiple regression model (MRM) and redundancy analysis (RDA) based on the ‘relaimpo’ and ‘vegan’ packages [30,32]. The effect of phytochemical compounds with significant differences among the varieties on soil microbial composition were analyzed using RDA on the ‘vegan’ package. A Procrustes analysis was used to analyze the relationships between microbial community compositions and metabolites using the ‘vegan’ package. The R software used in this work was R v. 4.3.3.

## 3. Results

### 3.1. Plant and Rhizosphere Soil Properties

The highest and lowest values of the tree growth indices (DBH and height) were observed in Q5 and Q4, respectively (Table 1). SOC values were significantly greater in the offspring (Q1~Q5) than in the hybrid parents (I101 and 84K), but showed no difference among the offspring. The TN content significantly differed among varieties, but the NO_3_^–^ and NH_4_^+^ contents showed no difference. TP and AP contents significantly differed among the varieties. The soil stoichiometric characteristics showed significant differences, with the highest C–N, C–P and N–P ratios observed at 84K, Q2 and Q4, respectively.

### 3.2. Rhizosphere Microbial Community Composition and Diversity

**Figure 1:**
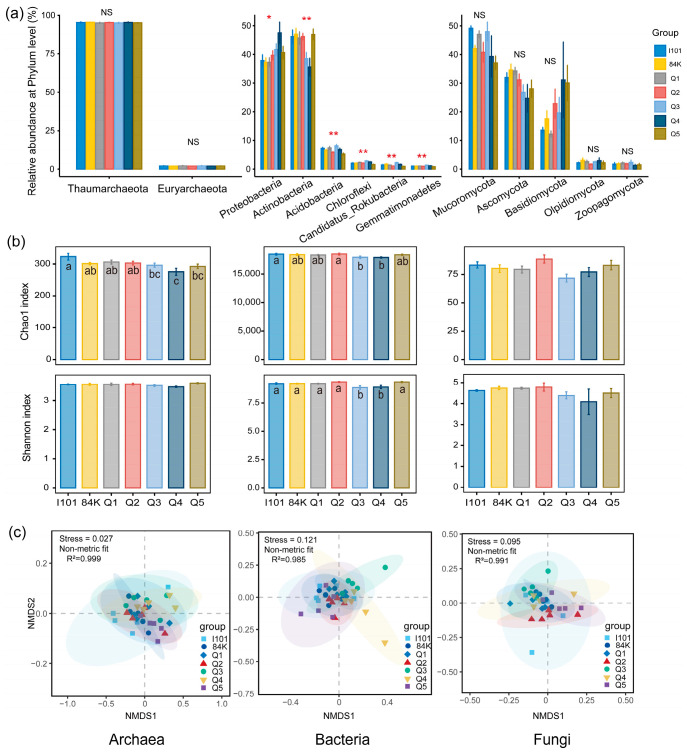
Comparison of metagenomic sequencing data from the rhizosphere soils of different poplar varieties. (**a**) Relative abundance of dominant microbial phyla; ** (*p* < 0.01) and * (*p* < 0.05) indicate significant differences based on a one-way ANOVA followed by an LSD test; NS: not significant. (**b**) Variations in bacterial, archaeal and fungal α-diversity (Shannon–Wiener and Chao1 indices); Different letters indicate a significant difference (*p* < 0.05) based on a one-way ANOVA followed by an LSD test. (**c**) Microbial β-diversity was analyzed using nonmetric multidimensional scaling (NMDS) based on Bray–Curtis distances.

After quality filtering, assembly and annotation, the high-quality sequences were clustered into 5 domains, 11 kingdoms, 217 phyla, 1384 families, 4336 genera and 27,323 species. The microbial community compositions were dominated by bacteria (98.31%), archaea (1.48%) and fungi (0.02%) at the kingdom level. The archaeal community was dominated by Thaumarchaeota and Euryarchaeota, which showed no differences in abundance among the varieties (Figure 1a). The bacterial community was dominated by Actinobacteria, Proteobacteria, Acidobacteria, Chloroflexi, Candidatus_Rokubacteria and Gemmatimonadetes, the abundance of which significantly differed among the varieties. The fungal community was dominated by Mucoromycota, Ascomycota and Basidiomycota, the abundance of which showed no differences among the varieties. The archaeal and bacterial Chao1 indices significantly differed among the varieties, with lower values in Q4. Only the bacterial Shannon–Wiener index significantly differed, with lower values in Q3 and Q4 (Figure 1b). NMDS was used to identify rhizosphere microbial community β-diversity at the species level. The archaeal (stress = 0.027), bacterial (stress = 0.121) and fungal (stress = 0.095) community compositions significantly varied among varieties (Figure 1c). 

**Figure 2:**
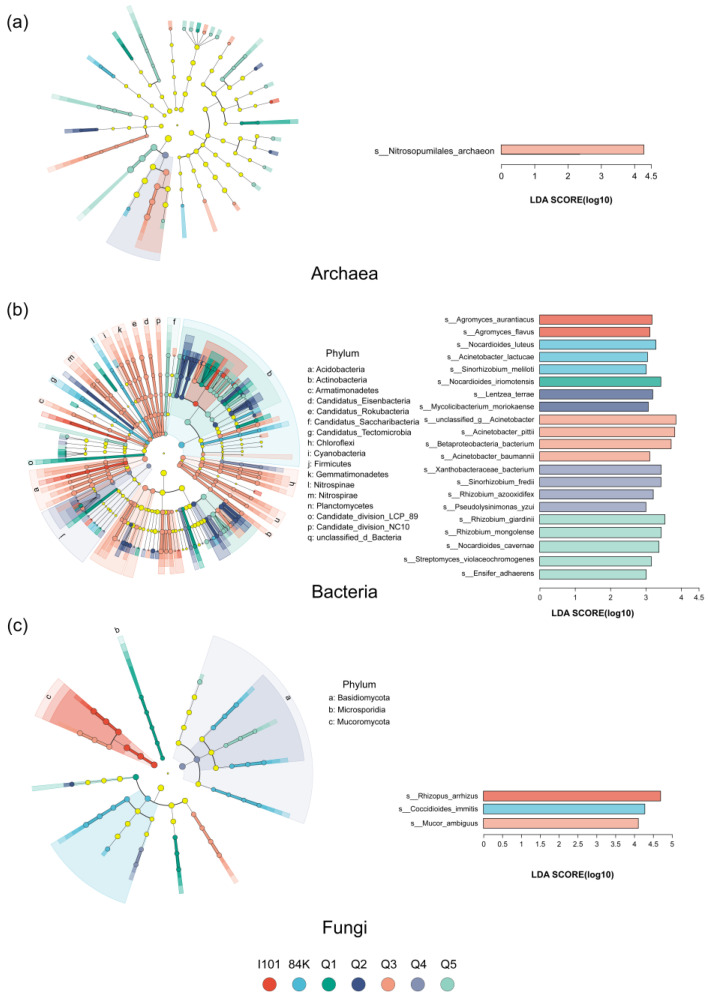
Linear discriminant analysis effect size (LEfSe) analysis of archaeal (**a**), bacterial (**b**) and fungal (**c**) communities among hybrid parents and offspring. Linear discriminant analysis (LDA) was used to identify the variations in species among the poplar varieties. Differently colored nodes in the figure indicate significant enrichment in the corresponding variety and yellow nodes indicate no significant difference among varieties.

A linear discriminant analysis effect size (LEfSe) analysis was used to identify the differences in microbes from the phylum level to the species level and microbial clades were identified based on linear discriminant analysis (LDA) scores. In total, 51 archaeal clades were selected based on LDA scores > 2.5 and *p* < 0.05, and *Nitrosopumilales_archaeon* (LDA scores > 2 and relative abundance > 1%) was enriched in Q3 (Figure 2a). A total of 296 bacterial clades were selected based on LDA scores > 2.5 and *p* < 0.05, and 5 bacterial species (LDA scores > 3 and relative abundance > 0.1%) were enriched in Q5 (Figure 2b). A total of 48 fungal clades were selected based on LDA scores > 2.5 and *p* < 0.05, and *Rhizopus_arrhizus*, *Mucor_ambiguus* and *Coccidioides_immitis* (LDA scores > 2.5 and relative abundance > 1%) were enriched in I101, 84K and Q3 at the species level, respectively (Figure 2c).

### 3.3. Rhizosphere Microbial Community Structure and Function

**Figure 3:**
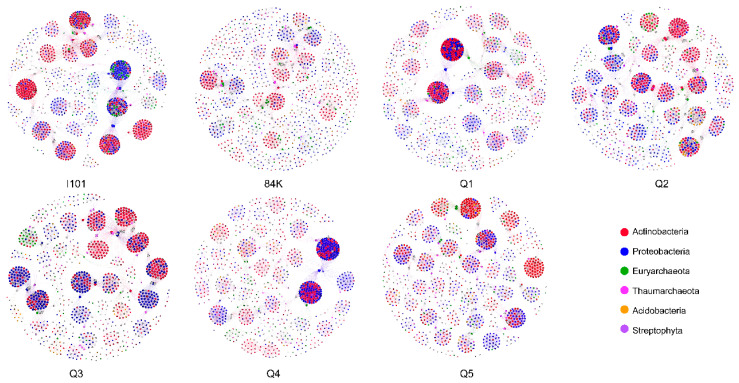
Co-occurrence networks of rhizosphere microbial communities in hybrid parents and their offspring. The nodes with different colors in the microbial networks represent the corresponding phyla.

Multiple network topological metrics consistently showed that the rhizosphere microbial co-occurrence patterns markedly differed between the hybrid parents and their offspring (Figure 3 and Table 2). The most complex and simplest co-occurrence networks were observed in I101 and 84K based on the values of the edge and the edge-to-node ratio, respectively. The co-occurrence networks showed a modular structure with modularity values > 0.5 for all varieties (Table 2). The percentages of positive correlations between nodes were greater than 80% in I101, Q2 and Q3 and less than 70% in 84K, Q1, Q4 and Q5.

**Figure 4:**
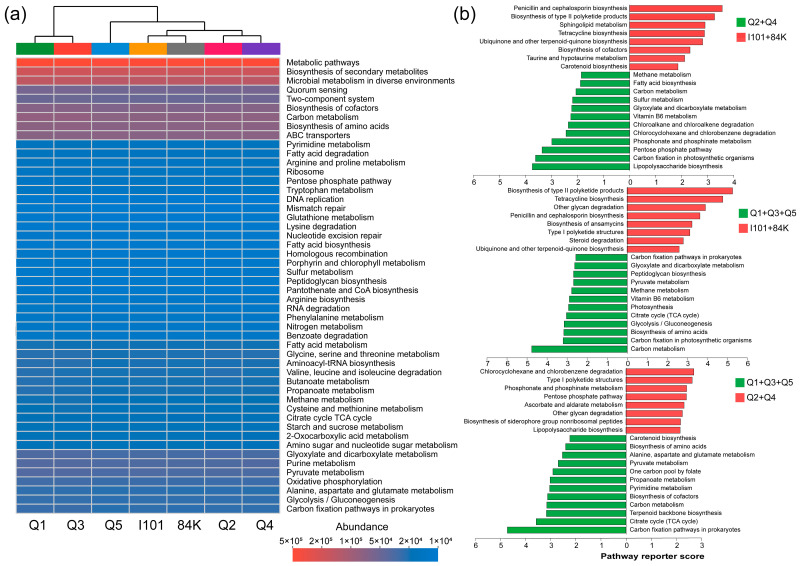
Heatmap cluster analysis of the top 50 rhizosphere microbial KEGG pathways at pathway level 3 (**a**) and the top 20 enriched KEGG pathways of different clustered groups (**b**).

We analyzed the similarity of the top 50 microbial functional groups based on KEGG pathway level 3 using a heatmap analysis and hierarchical cluster analysis (Figure 4a). The five most abundant KEGG pathways were metabolic pathways, biosynthesis of secondary metabolites, microbial metabolism in diverse environments, carbon metabolism and biosynthesis of amino acids. Poplar varieties were clustered into three groups, i.e., I101 and 84K; Q2 and Q4; and Q1, Q3 and Q5 (Figure 4a). We analyzed the enrichment of KEGG pathway level 3 among the clustered groups and the top 20 pathways with reporter score values > 1.65 were listed. Compared with those in the group of hybrid parents (I101 and 84K), 12 pathways were enriched in the clustered groups of Q2 and Q4 and the clustered groups of Q1, Q3 and Q5, respectively. (Figure 4b).

**Figure 5:**
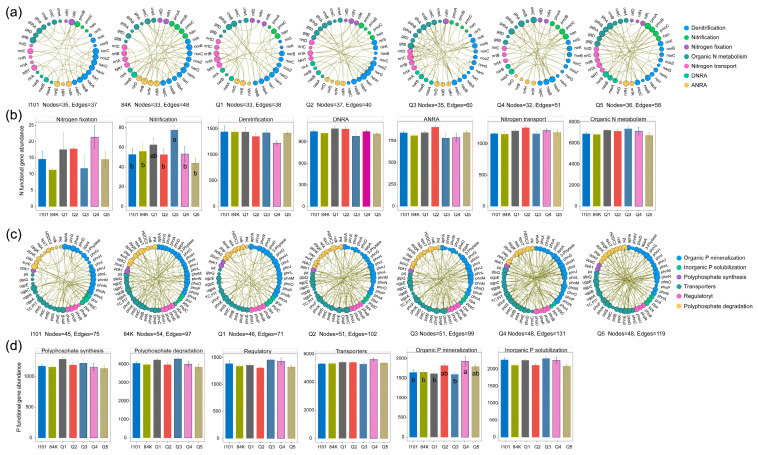
Co-occurrence networks and abundance of microbial functional genes associated with the N (**a**,**b**) and P (**c**,**d**) cycles of different varieties. Different letters in (**b**,**d**) indicate a significant difference (*p* < 0.05) based on a one-way ANOVA followed by an LSD test.

Furthermore, we analyzed the differences and co-occurrence networks of microbial functional genes associated with the N and P cycles. Higher network edge values of N cycles were observed in Q3 and Q5, and the functional gene abundance of nitrification significantly differed among varieties, with the highest value in Q3 (Figure 5a,b). Similarly, higher network edge values of P cycles were observed in Q4 and Q5, and the abundance of functional genes related to organic P mineralization significantly differed among the varieties, with the highest value occurring in Q4 (Figure 5c,d).

### 3.4. Rhizosphere Untargeted Metabolomics

**Figure 6:**
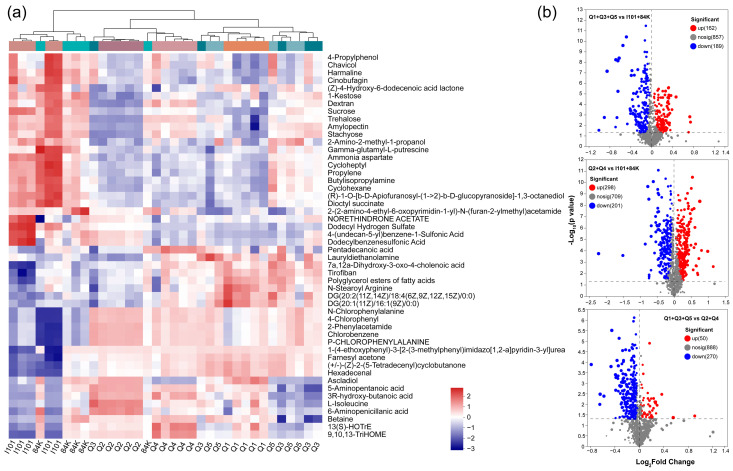
Heatmap cluster analysis of the top 50 rhizosphere metabolites based on relative expression (**a**) and volcano plots of differentially expressed metabolites (**b**). The volcano plot depicts the log2-fold change on the *x*-axis and the significant differences in the metabolites (−log_10_ *p*-value) are shown on the *y*-axis.

A total of 1208 individual metabolites were detected and then classified based on the KEGG database. We analyzed the similarity of the top 50 metabolites based on the relative transcript level using a heatmap analysis and hierarchical cluster analysis. Similar to the microbial functional gene clustering results, the poplar varieties were clustered into three groups, i.e., I101 and 84K; Q2 and Q4; and Q1, Q3 and Q5 (Figure 6a). Compared with those in the hybrid parents (I101 and 84K), the levels of 162 and 189 metabolites increased and decreased, respectively, in the group of Q1, Q3 and Q5, and those with 298 and 201 metabolites increased and decreased, respectively, in the group of Q2 and Q4. Compared with those in the group of Q2 and Q4, the levels of 50 and 270 metabolites significantly increased and decreased in the group of Q1, Q3 and Q5, respectively (Figure 6b).

**Figure 7:**
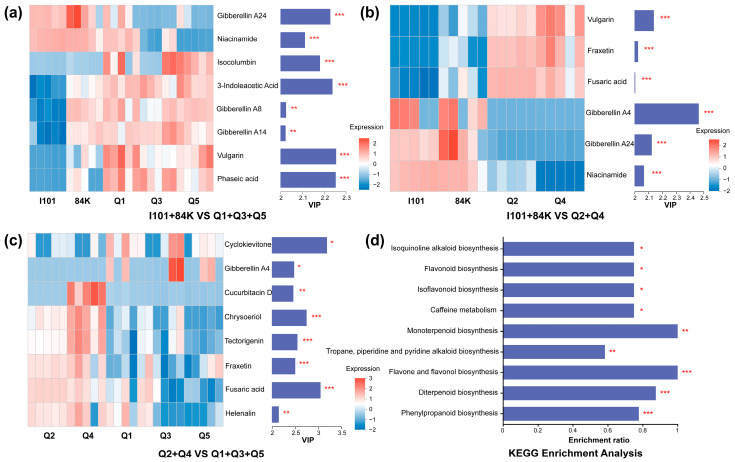
Variable importance in projection (VIP) scores of phytochemical compounds based on the PLS-DA model. Heat maps with red and blue colors reveal the relative expression of the metabolites among different clustered groups of poplar varieties (**a**–**c**). The metabolites with VIP > 2 were selected. Enrichment analysis of KEGG pathways associated with phytochemical compounds among poplar varieties (**d**). *** (*p* < 0.001), ** (*p* < 0.01) and * (*p* < 0.05).

In this study, 146 phytochemical compounds were selected from the poplar varieties. Compared with those in the group of hybrid parents (I101 and 84K), the levels of phaseic acid, vulgarin, gibberellin A8, gibberellin A14, 3-indoleacetic acid and isocolumbin increased and niacinamide and gibberellin A24 decreased in the group of Q1, Q3 and Q5, respectively (Figure 7a). Compared with those in the hybrid parents (I101 and 84K), the levels of vulgarin, fraxetin and fusaric acid increased and gibberellin A4, gibberellin A24 and niacinamide decreased in the group of Q2 and Q4, respectively (Figure 7b). Compared with those in the group of Q2 and Q4, the levels of helenalin, fusaric acid, fraxetin, tectorigenin and cucurbitacin D decreased and gibberellin A4 and cyclokievitone increased in the group of Q1, Q3 and Q5, respectively (Figure 7c). In total, 9 KEGG pathways associated with phytochemical compounds were enriched and the enrichment ratios of pathways ≥ 1 were monoterpenoid biosynthesis and flavone and flavonol biosynthesis (Figure 7d).

### 3.5. Effects of Soil Nutrients and Metabolites on the Microbial Community

**Figure 8:**
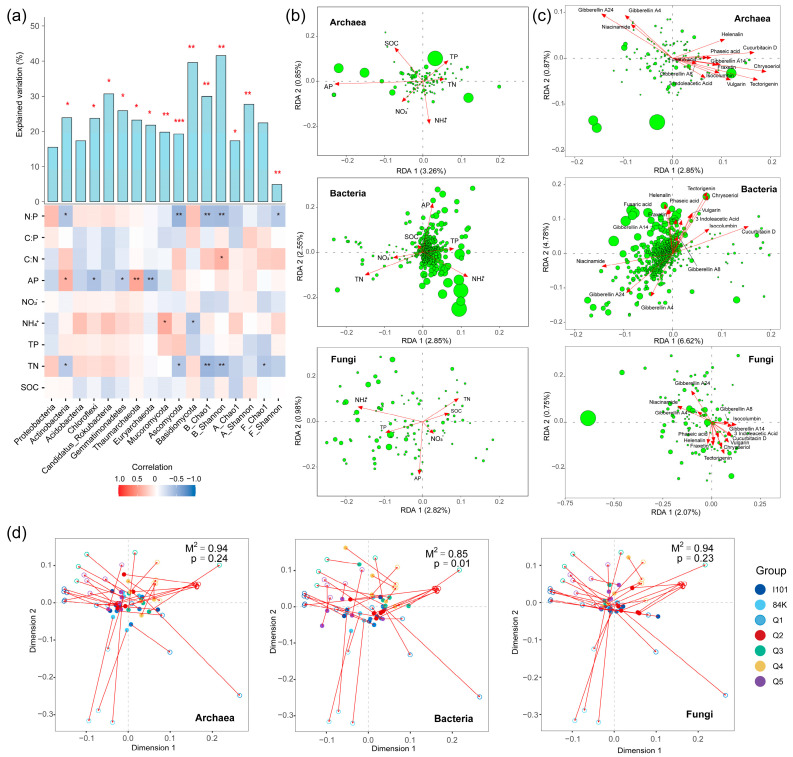
Effects of soil nutrients, phytochemical compounds and metabolites on microbial community composition. Effects of soil nutrients on microbial dominant phyla based on a multiple regression model (**a**). RDA was used to explore the influence of soil nutrients (**b**) and phytochemical compounds (**c**) on rhizosphere archaeal, bacterial and fungal community compositions. Relationships between metabolites and microbiomes were determined using a Procrustes analysis (**d**). *** (*p* < 0.001), ** (*p* < 0.01) and * (*p* < 0.05).

Multiple regression was used to estimate the relationships between soil nutrients and the dominant phyla. Soil nutrients explained more than 30% of the variation in the phyla Candidatus_Rokubacteria and Basidiomycota and were significantly correlated with the dominant microbial phyla, excluding Proteobacteria and Acidobacteria (Figure 8a). The major bacterial and archaeal phyla were significantly correlated with soil AP. The Shannon–Wiener and Chao1 indices of the bacterial and fungal communities were sensitive to changes in the TN and N–P ratios. RDA was used to explore the effects of soil nutrients and 15 phytochemical compounds with significant difference in the microbial community composition at the species level. Soil nutrients explained only 8.48%, 5.53% and 6.11% of the total variation in the bacterial, archaeal and fungal community compositions, respectively (Figure 8b). The 15 phytochemical compounds explained 18.24%, 7.15% and 6.68% of the total variation in the bacterial, archaeal and fungal community compositions, respectively (Figure 8c). The correlation between metabolites and microbial community composition was estimated by a Procrustes analysis. The effects of rhizosphere metabolites on the bacterial community composition were significant (M^2^ = 0.85; *p* = 0.01), but those on the archaeal (M^2^ = 0.94; *p* = 0.24) and fungal (M^2^ = 0.94; *p* = 0.23) communities were not significant (Figure 8d).

## 4. Discussion

The rhizosphere microbiome is recognized as an extension of the plant root phenotype and plays key roles in plant adaptations to changes in the internal and external environment [4,6]. Various studies have reported that the rhizosphere microbiome can directly impact plant functional traits and economic strategies [5,33]. In contrast, plants have a decisive effect on rhizosphere microbiomes and can recruit specific microorganisms by releasing root exudates [5,14,15,34,35]. Therefore, improving ecological plant–microbiome interactions has become a new avenue for plant breeding [2]. The effects of breeding selection on the rhizosphere microbiome of offspring have been mostly studied in crop breeding and studies on woody plants are limited [8].

The same ecological site and forest management measures ensured that variations in rhizosphere microbiomes were mainly driven by the breeding of poplar hybrids. Rhizosphere soil microbiomes and metabolites differed between hybrid parents and their offspring, indicating that microbial functional genes and metabolites were both clustered into the following three groups: I101 and 84K; Q2 and Q4; and Q1, Q3 and Q5. These findings confirmed the key controls of rhizosphere metabolites on the microbiome. The observation that the Q1, Q3 and Q5 group was separated from the Q2 and Q4 group supported the important effect of intraspecific genotypic variations on the phylogenetic composition of rhizosphere microbial communities [16]. At least a part of the variations in the rhizosphere microbial community compositions among hybrid parents and offspring may be caused by plant-associated microorganisms, which can be selected by the plant genotype [2,8,36,37].

Breeders reported that breeding selection imposes differential selective pressure on the plant-root-associated microbiome [8]. Although the rhizosphere microbial β-diversity significantly differed among the parents and offspring, only bacterial Chao1 and the Shannon–Wiener indices significantly differed. The dominant archaeal and fungal phyla showed no difference among the parents and offspring, but the dominant bacterial phyla significantly differed. In combination with the finding that 5.49%, 14.90% and 7.86% of archaeal, bacterial and fungal community compositions significantly differed among the varieties, respectively, on the basis of the LEfSe analysis, we suggest that breed selection imposes greater selective pressure on the rhizosphere bacterial community.

Soil microorganisms can form complex interaction networks based on niche spaces shared and competed for by microbial community members [38]. The edge-to-node ratio can reflect the closeness of the connections between microorganisms in the microbial network and the percentage of positive and negative correlations in the network can reflect resource-sharing and competition [18]. The edge-to-node ratios and positive-to-negative ratios of the microbial networks for the hybrid offspring were between those of the parents. This finding suggests that the complexities of rhizosphere microbial co-occurrence networks in offspring are likely to be affected by their parents.

Untargeted metabolomics allows us to identify the compounds that are secreted by roots and microbiomes in the rhizosphere [17]. Recent studies have reported that untargeted metabolomics can be used to assess changes in microbial function and drive rhizosphere microbial community assembly [39,40]. When all of the rhizosphere metabolites were considered, approximately 26.49~41.31% of the total metabolites differed among three clustered groups and those differentially abundance metabolites may have acted as nutrient sources and signals to recruit specific rhizosphere microflora [5,15]. The high levels and different types of carbon sources in the rhizosphere metabolites acted as chemoattractants that directly affected the bacterial community composition (Fig. 8d), resulting in significant difference in dominant bacterial phyla among parents and offspring. Furthermore, the huge influence of rhizosphere metabolites on dominant bacterial flora can help us to understand the variations in bacterial β-diversity (stress=0.121 > 0.1) among the parents and offspring.

Interactions between plant and rhizosphere microbiomes are mostly achieved via root exudates, which contain an array of primary and secondary plant metabolites and are mainly influenced by interactions between the substrate and the plant genotype [10,17,41]. Plants can regulate rhizosphere microbiomes by providing an energy-rich environment and producing a wide variety of signals [11]. We analyzed the differences in the VIP scores of phytochemical compounds to explore the effects of plant genotypes on the plant metabolites. 3-indoleacetic acid, gibberellin and phaseic acid, which are associated with plant growth and resistance, were enriched in the Q1, Q3 and Q5 group. Fraxetin, associated with Fe (III) mobilization, was enriched in the Q2 and Q4 group compared with the hybrid parent group. These phytochemical compounds, which significantly differ among poplar varieties, explained 7.15%, 18.24% and 6.68% of the total variation in the archaeal, bacterial and fungal community compositions, suggesting that plant metabolites exert key control over the rhizosphere microbial community composition [10].

Microbial functional genes in the rhizosphere are determined mainly by trophic relationships and interactions between microbiomes and plants [42]. Terpenoids and flavonoids play important role in plant growth and defense as well as the interaction of plants with their environment [43,44]. The KEGG pathways of monoterpenoid biosynthesis, diterpenoid biosynthesis, isoflavonoid biosynthesis and flavone and flavonol biosynthesis were enriched based on the analysis of phytochemical compounds (Figure 7d). Compared with the microbial functional genes in the hybrid parent group, the KEGG pathways associated with the citrate cycle, carbon metabolism, propanoate metabolism and pyruvate metabolism were enriched in the Q1, Q3 and Q5 group and the pathways of phosphonate and phosphinate metabolism, pentose phosphate pathway and lipopolysaccharide biosynthesis were enriched in the Q2 and Q4 group. The mechanism of the variations in the enriched pathways of microbial functional genes between two offspring groups is highly complex and enriched phytochemical compounds may play important roles in regulating rhizosphere microbial functional groups [2,8,37].

Rhizosphere microbiomes can modulate key steps in nutrient cycles. leading to intraspecific variations in the plant nutrient status [16]. In this study, we focused on the analysis of functional genes involved in the N and P cycles, which play key roles in plant economic strategies [5]. The complexity of the networks in the N-related functional genes varied among the hybrid offspring and the most simple and complex networks were observed in the Q1 and Q3 groups, respectively. This finding could be partly explained by the variations in root exudates, which may have enhanced the mobilization and solubilization of mineral-associated organic matter and subsequently affected nitrogen availability [10,45]. Only nitrification- and organic-P-mineralization-related functional genes significantly differed among the hybrid parents and offspring, suggesting that the variations in microbial functional groups associated with N and P cycles were dominated by several key functional genes.

Although strong correlations between dominant microbial phyla and soil nutrients were observed, soil nutrients explained limited variations in the archaeal (5.53%), bacterial (8.48%) and fungal (6.11%) community compositions at the species level. The first two RDA axes of the bacterial, archaeal and fungal communities were associated with AP, confirming the important control effects of phosphorus availability on the rhizosphere microbial community [46]. Similarly, soil nutrients explained 4.93%, 8.5% and 5.05% of the total variation in C, N and P functional genes, respectively. These observations confirmed that rhizosphere metabolites associated with plant genotype, rather than soil nutrients, drive changes in rhizosphere microbiomes under the same ecological conditions.

## 5. Conclusions

Rhizosphere soil microbial community compositions, structure and function significantly differed among poplar hybrid parents and offspring. Breed selection imposed greater selection pressure on the poplar rhizosphere bacterial community than on archaeal and fungal communities. Rhizosphere microbial functional genes were clustered into three groups; this was consistent with the metabolomic classification. The effects of metabolites on rhizosphere archaeal and fungal community compositions were not significant and the variations in bacterial community composition were mainly driven by metabolites. Phytochemical compounds played important roles in regulating rhizosphere bacterial community compositions. Variations in microbial functional groups associated with N and P cycles were dominated by several key functional genes.

## Figures and Tables

**Table 1:** Tree growth properties and rhizosphere soil nutrients.

Properties	I101	84K	Q1	Q2	Q3	Q4	Q5	F	*p*
Height (m)	12.37 ± 0.45 ab	11.15 ± 0.58 bc	13.42 ± 1.15 a	13.05 ± 1.03 a	13.42 ± 1.56 a	10.45 ± 1.67 c	13.95 ± 1.25 a	4.852	0.003
DBH (cm)	13.4 ± 0.91 b	13.25 ± 0.52 b	13.53 ± 0.5 b	13.67 ± 1.09 b	13.15 ± 1.13 b	10.3 ± 1.24 c	16.15 ± 1.05 a	10.588	<0.001
SOC (g kg^−1^)	4.15 ± 1.33 b	5.56 ± 1.74 ab	6.51 ± 0.94 a	7.14 ± 0.99 a	6.66 ± 0.54 a	7.03 ± 0.68 a	6.86 ± 1.98 a	3.39	0.012
TN (g kg^−1^)	1.21 ± 0.23 bc	1.0 ± 0.19 cd	0.90 ± 0.21 d	1.01 ± 0.12 cd	1.43 ± 0.30 ab	1.66 ± 0.15 a	1.29 ± 0.07 b	9.939	<0.001
TP (g kg^−1^)	0.92 ± 0.05 a	0.91 ± 0.12 a	0.93 ± 0.08 a	0.77 ± 0.13 b	0.83 ± 0.07 ab	0.91 ± 0.05 a	0.93 ± 0.06 a	2.863	0.026
NO_3_^−^ (mg kg^−1^)	4.97 ± 0.62	5.23 ± 1.05	4.07 ± 1.76	4.62 ± 2.1	4.23 ± 1.18	4.74 ± 1.14	2.85 ± 1.28	1.825	0.129
NH_4_^+^ (mg kg^−1^)	8.72 ± 2.35	7.67 ± 1.79	7.1 ± 1.18	7.25 ± 2.89	7.77 ± 1.99	6.22 ± 1.47	5.8 ± 2.68	1.109	0.381
AP (mg kg^−1^)	12.9 ± 0.8 b	14.25 ± 0.53 a	13.99 ± 1.85 a	13.21 ± 0.93 ab	12.21 ± 1.23 b	12.16 ± 0.59 b	13.45 ± 0.66 ab	3.138	0.017
C–N	3.60 ± 1.66 d	5.64 ± 1.75 bc	7.43 ± 1.1 a	7.11 ± 1.02 ab	4.82 ± 1.12 cd	4.23 ± 0.21 cd	5.33 ± 1.64 cd	5.731	<0.001
C–P	4.57 ± 1.55 c	6.03 ± 1.36 bc	7.13 ± 1.69 b	9.50 ± 1.70 a	8.11 ± 0.82 ab	7.73 ± 0.83 ab	7.46 ± 2.45 ab	4.713	0.002
N–P	1.32 ± 0.24	1.11 ± 0.24	0.99 ± 0.32	1.35 ± 0.23	1.73 ± 0.26	1.83 ± 0.18	1.39 ± 0.11	8.485	<0.001

Values are the means ± standard deviation (n = 5); different letters indicate a significant difference (*p* < 0.05) based on a one-way ANOVA followed by an LSD test.

**Table 2:** Topological metrics of the rhizosphere microbiome.

Topological Metrics	I101	84K	Q1	Q2	Q3	Q4	Q5
Nodes	1124	1104	1105	1114	1066	1033	1132
Edges	24,351	10,842	20,896	18,523	18,981	18,584	17,270
Edges/nodes	21.66	9.82	18.91	16.63	17.81	17.99	15.26
Modules	19	19	26	22	23	19	31
Modularity	0.86	0.84	0.82	0.87	0.86	0.82	0.89
Transitivity	0.95	0.74	0.96	0.93	0.92	0.97	0.94
Density	0.039	0.018	0.034	0.030	0.033	0.035	0.027
Diameter	27.29	30.25	25.34	25.34	27.29	21.44	35.08
Positive correlation (%)	86.44	61.67	69.77	87.34	83.31	74.74	68.91
Negative correlation (%)	13.56	38.33	30.23	12.66	16.69	25.26	31.09

## Data Availability

The original contributions presented in the study are included in the article, further inquiries can be directed to the corresponding author.
